# Bacterial biofilm in salivary stones

**DOI:** 10.1007/s00405-019-05445-1

**Published:** 2019-04-26

**Authors:** Ramón Perez-Tanoira, Antti Aarnisalo, Aaro Haapaniemi, Riitta Saarinen, Pentti Kuusela, Teemu J. Kinnari

**Affiliations:** 1 Department of Otorhinolaryngology–Head and Neck Surgery, Helsinki University Hospital, University of Helsinki, Haartmaninkatu 4 E, 00029 HUS Helsinki, Finland; 2 Department of Infectious Diseases, IIS-Fundación Jiménez Díaz, Madrid, Spain; 3 Department of Bacteriology and Immunology, Haartman Institute, Helsinki University Hospital, and University of Helsinki, Helsinki, Finland

**Keywords:** Salivary stones, Biofilm, Sialadenitis, Sialolithiasis, Sialendoscopy

## Abstract

**Purpose:**

To assess the susceptibility of salivary stones to bacterial biofilm formation, which may be involved in the development of salivary gland infection, and to investigate a relation between microbiological aspects and patient characteristics.

**Methods:**

This prospective study comprises of 54 patients with sialolithiasis attended in Helsinki University Hospital during 2014–2016. A total of 55 salivary stones were removed, and studied for biofilm formation using fluorescence microscopy and sonication. The isolated organisms were quantified and identified using matrix-assisted laser desorption ionization time-of-flight mass spectrometry.

**Results:**

Biofilm formation was confirmed on the surface of 39 (70.9%) stones. A total of 96 microorganisms were isolated from 45 salivary stones (81.8%). Two or more organisms were isolated in 33 (73.3%) cases. The main isolates were *Streptococcus mitis/oralis* (*n* = 27; 28.1%), followed by *Streptococcus anginosus* (*n* = 10; 9.6%), *Rothia* spp. (*n* = 8; 8.3%), *Streptococcus constellatus* (*n* = 7; 7.3%), and *Streptococcus gordonii* (*n* = 6; 6.2%). In all patients showing pre-operative (12 cases) or peri-operative (three cases) drainage of pus, the presence of biofilm was detected in microscopy (*p* = 0.004). Four patients showed post-operative infection, and in three of them (75.0%), the presence of biofilm was detected. Increased number of pus drainage was found among patients with reflux symptoms or use of proton-pump inhibitors.

**Conclusions:**

Salivary stones are susceptible to bacterial biofilm formation, which could be related with the development and severity of the inflammation and the refractory nature of the disease. Sonication of salivary gland stones could be a useful method for finding the etiology of the chronic infection.

## Introduction

Bacterial growth in the form of biofilm has been associated with most ear, nose, and throat infections [1, 2]. In particular, implanted biomaterials and other passive surfaces with poor host defense, such as salivary calculi, are prone to bacterial attachment and biofilm formation. The biofilm is a complex slimy mass of bacteria and extracellular components, in which the bacterial community lives in metabolically inactive form relatively isolated from its surroundings. Both antibiotics and host defense have limited effect on the bacteria in biofilm. The eradication of biofilm from passive surfaces is demanding if not impossible. In clinical settings, this often results in retrieving infected implants.

Biofilm growth has been associated with chronic otitis media and mastoiditis as well as chronic infections of the adenoid tissue. Planktonic bacteria may disperse from mature biofilm and cause an acute phase of the infection. This is typical in mild chronic diseases or recalcitrant infections such as recurrent and chronic otitis media [3].

Biofilm is found in natural surfaces contacting water. The most typical form is slippery slime on passive materials such as river stones. In human body, the most typical manifestation is dental plaque, which is also related to the formation of dental calculus. Organic substances including calcium phosphates may precipitate in the biofilm plaque forming a calcification on the tooth surface [4]. The calcification of the dental plaque is more prominent in the immediate vicinity of the excretory ducts of the large salivary glands in the lingual surfaces of the lower jaw and the buccal surfaces of the upper jaw where higher concentrations of salivary calcium precipitate on the biofilm [5, 6].

Sialolithiasis, the formation of stones in the salivary duct or gland, is a relatively common disease occurring in 0.1–1% of the population [7]. It mainly affects the submandibular glands, which harbor up to 90% of salivary stones [8]. The main etiological factors for stone formation are related to saliva retention and saliva composition: among sialolithiasis patients, the salivary calcium concentration is higher compared to healthy individuals and the concentration of crystallization-inhibiting phytate has been shown to be lower [9]. The submandibular ductal system is more prone to sialolith formation, since it has a long-and-winding passage, and its saliva has higher viscosity and calcium phosphate content than the parotid saliva [7]. Other risk factors are mainly related to increased saliva viscosity, which may result from chronic dehydration typically seen in elderly persons or to secretory inactivity caused by anticholinergic medication, some systemic diseases such as Sjögren’s syndrome, or irradiation therapy [10].

The stone grows gradually on a small core. However, the origin of this nidus is still unknown. According to current theory, the calculus forms around a particle which is either a foreign body, desquamated epithelial cells, high viscosity mucins, or other particles brought to the ductal system. According to a retrograde migration theory, these particles are bacteria or microbial colonies. The role of microbials or biofilm as the prime mover is yet to be proved. A strong consensus, however, exists that there is a nidus of unknown origin, and thereafter, in a timely process, a series of layers accumulate on it. The inorganic layer components are calcium carbonate and calcium phosphate in an apatite structure. The organic layers consist of glycoproteins, mucopolysaccharides, and cell detritus [5].

Saliva under normal circumstances is sterile until it leaves the salivary duct and enters the oral cavity [11]. The microbial diagnostics in oral cavity are always complicated as the saliva in mouth is contaminated with the oral microbiome. In addition, salivary stones retrieved through the mouth are practically always contaminated. Thus, it is not surprising that the most detected bacterial species in sialolithiasis belong to the streptococcus genus, part of the oral microbiome [5]. There is limited literature considering the bacterial biofilm in salivary stones. Fusconi et al. have recently shown biofilm-type bacterial aggregates in the core of salivary stones surrounded by organic matrix with glycoprotein nature suggesting the presence of biofilm in the mature salivary stones [12]. One of the most interesting questions is whether the presence of oral microbials and biofilm in salivary ducts happens prior to stone formation, as suggested in the theory of the retrograde migration, or as a result of secondary contamination of the stone.

In this study, we have investigated the microbiology of salivary stones collected from a series of prospective cases of sialolithiasis in the submandibular and parotid glands. The goal was to study the clinical manifestation and risk factors related to salivary stones as well as to relate these details to the microbiology of the stones. We used matrix-assisted laser desorption ionization time-of-flight mass spectrometry (MALDI-TOF) analysis to investigate the microbials isolated from the stones. Furthermore, biofilm presence was studied using fluorescence microscopy.

## Materials and methods

### Patients

Stones were collected prospectively from 54 patients, 30 females (55.6%) and 24 males (44.4%), operated at the Department of Otorhinolaryngology–Head and Neck Surgery, Helsinki University Hospital, Helsinki, Finland. The salivary stones were collected from May 2014 to May 2015. However, due to their rarity, the collection of parotid stones continued until August 2016.

In sialendoscopy, the papilla region was first anesthetized with a topical spray of 10 mg/ml lidocaine (Xylocain®) and a small amount of 10 mg/ml lidocaine with adrenalin (Lidocain®) was infiltrated under the papilla. Next, the papilla was dilated with dilators to allow the passage of a 1.1 mm or 1.3 mm Storz all-in-one sialendoscope. During sialendoscopy, the ductal system was irrigated with 0.9% saline solution to maintain visibility. In endoscopic stone removal, stones were captured in a Dormia basket passed via the working channel of the scope and pulled out through the duct with or without the aid of a small papillotomy. In case of transmucosal removal, a local anesthetic (lidocaine cum adrenalin) was first infiltrated in the incisional area in the floor of the mouth. Then, a mucosal incision was made and the duct with the stone identified. The stone was removed via an incision in the duct. In transcutaneous removal of stones from the parotid duct, the skin was first cleaned and prepped according to normal aseptic standards. An endoscopist was then visualized the stone via the duct. The transillumination effect was used to identify the location of the stone, and a second surgeon made an incision over the skin in the illuminated area, identified and incised the duct with the stone, and removed the stone.

There were 42 submandibular stones (76.4%) and 13 parotid stones (23.6%). Of the submandibular stones 40 were removed endoscopically or through an incision in the floor of the mouth, and two were removed in a sterile fashion during submandibulectomy. Ten of the parotid stones were removed endoscopically or using a combined technique and three through the skin in a sterile fashion without sialendoscopic assistance. All operations except the two submandibulectomies were done under local anesthesia.

Immediately after collection, the stones were stored in Eppendorf tubes and transported to the research laboratory for further analysis.

### Sample analysis

The removed stones were sectioned in two halves in a sterile environment in a biosafety cabinet. One half was prepared for the microscopy, and the other half was sonicated to analyze the bacteria and biofilm formation.

### Microscopy

The stone halves were washed three times with PBS, dried and thereafter stained for 2 min with a rapid fluorescence staining method using Acridine Orange (BD Diagnostics, Sparks, MD, USA). Images were taken with 20 × objective magnification using a Leica DM6000 B/M fluorescent microscope equipped with Leica DFC420 digital camera (Leica Microsystems, Wetzlar, Germany).

### Sonication

**Fig. 1:**
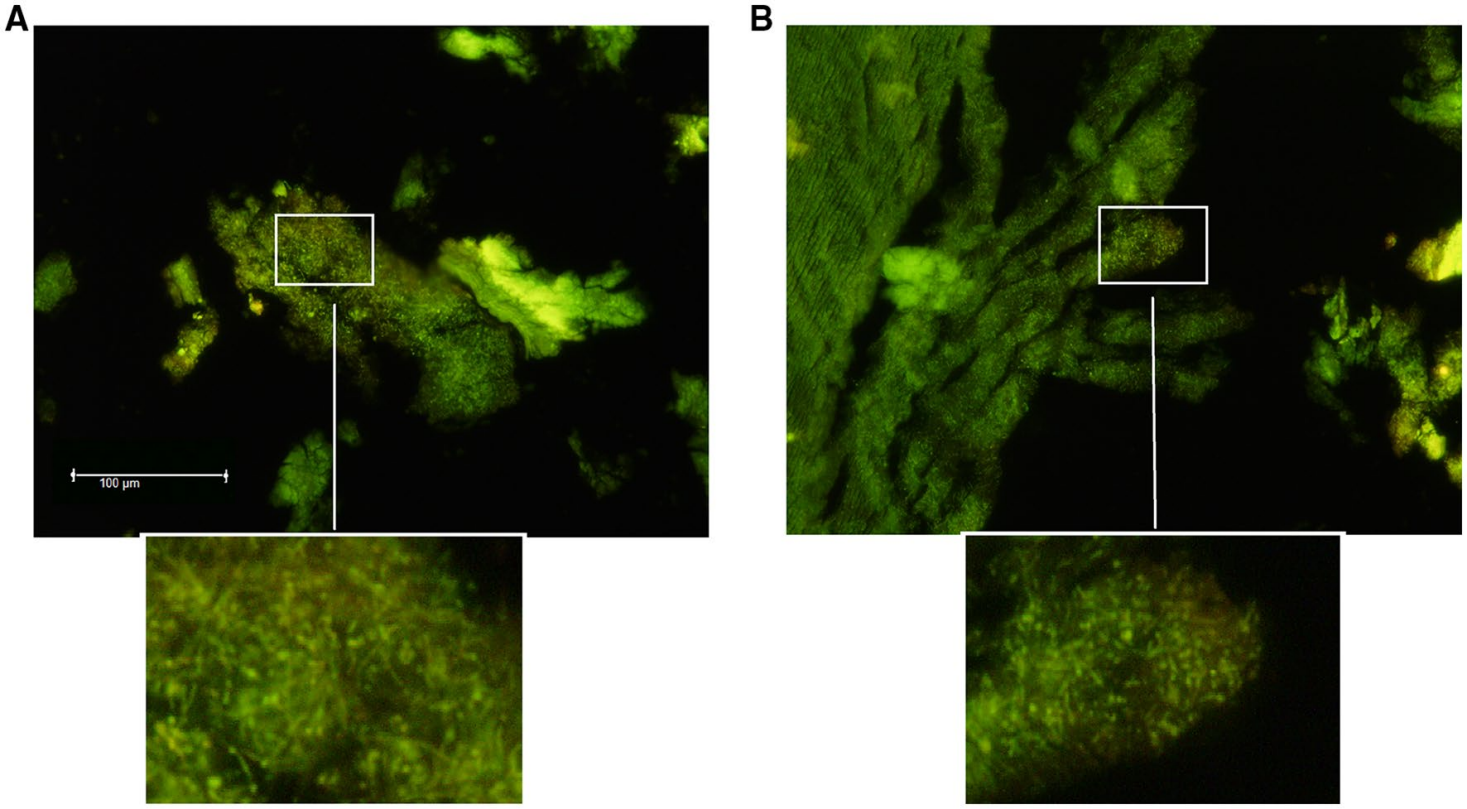
Representative fluorescent microscope images of the salivary stone showing adherent bacteria/biofilm. The samples A and B were stained with Acridine Orange (BD Diagnostics, Sparks, MD, USA). 20× magnification. Scale bar represents 100 µm

**Fig. 2:**
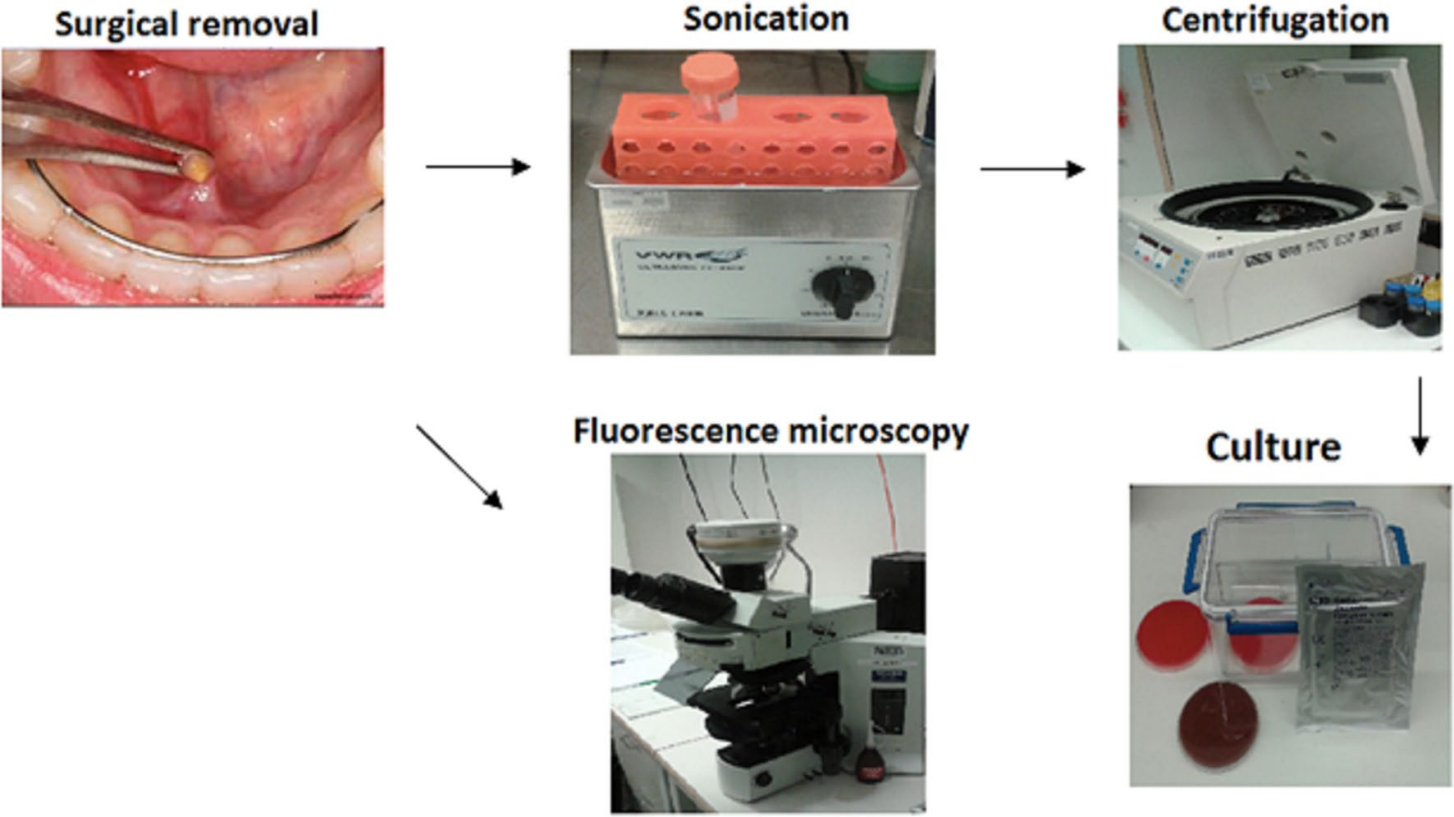
Scheme of procedure of salivary stones analysis

Stone halves were introduced aseptically in 2.5 mL of sterile phosphate buffer saline (PBS) (pH 6.8, BioMérieux, Marcy-l'Étoile, France) in Falcon tubes and thereafter sonicated for 5 min in an ultrasonic bath USC100T (VWR, Leuven, Belgium) at 45 kHz with a power output of 300 W, as described in Fig. 1 [13–15]. After that, the stone halves were measured to adjust the amount of bacteria to the actual surface area (Fig. 2).

## Bacterial cultures

The sonicate was centrifuged at 2500*g* for 10 min, and supernatant was then discharged. Sediment was re-suspended in 1.5 mL of PBS and vortexed, and then, 10 µL of the suspension was inoculated into each of the following culture media: Tryptic soy 5% sheep blood agar, chocolate agar and Schaedler 5% sheep blood agar. All media were incubated for 7 days at 37ºC under different conditions: 5% CO_2_ atmosphere (tryptic soy 5% sheep blood agar and chocolate agar) and anaerobic atmosphere (Schaedler 5% sheep blood agar). The media were checked daily for microbial growth, and the result was expressed quantitatively in colony-forming units (CFU)/mL (CFU/mL = CFU on the plate/10 µL × 1000 µL/1 mL = CFU on the plate × 100).

Total bacterial counts were adjusted to the actual surface area of the salivary stone, taking into account all different faces (total bacterial count = CFU/cm^2^).

Isolated organisms were identified by MALDI-TOF system (Vitek-MSª BioMérieux, Marcy-l’ Étoile, France).

### Ethics statement

The study protocol was approved by the Ethics Committee of the Helsinki University Hospital. All patients signed an informed consent. This study conforms to Declaration of Helsinki.

## Results

Table 1 shows the main epidemiological characteristics and the clinical manifestations related to sialadenitis of 54 patients. The mean age of the patients was 46.6 ± 18.2 years (mean ± SD, range 10–86 years). For the submandibular group (41 patients), the mean age was 42.6 ± 18.0 and 59.2 ± 12.3 years for the parotid group (13 patients) (*p* = 0.003 Mann–Whitney test). None of the patients had a manifest parodontitis.

A total of 55 stones were collected. In 44 cases, the stone was removed via the peroral route, in five cases through the skin and in six using a combined technique. The submandibular stones were removed via the peroral route in 40 cases (95.2%) and using the sterile transcutaneous route during a sialadenectomy in two cases (4.8%). On the other hand, of the 13 parotid stones four (31%) were operated via the peroral route, six (46%) using a combined technique, and three (23%) through the skin with a sterile technique.

Stones were located mainly in the main duct [29 (53%)], followed by the ductal hilus and papilla [11 (20%) and 10 (18%) respectively]. Four cases (7.3%) of intraglandular stones were found, and in one case (1.8%), there were several salivary stones located in the ductal, hilar, and intraglandular areas.

All patients had pre-operative symptoms, although, in many cases, they were quite mild. A total of 15 patients (27.3%), suffered from pus drainage (12 patients pre-operatively and three perioperatively), six from swelling (10.9%), and three patients each from fever and skin redness (5.5%). Four patients (7.3%) developed a post-operative infection. In two (50%) of these cases, the transcutaneous technique was used. Altogether, two out of the five (40%) cases with sterile extraction developed a post-operative infection (*p* = 0.003) (Table 1).

**Table 1:** Epidemiological factors associated with clinical manifestations and worse outcome for patients with salivary stones (total patients *n* = 54)

	*n* (%)	SR* n* = 3	Fever* n* = 3	PD* n* = 15	PI* n* = 4
Age > 50 years	27 (50.0%)	3	2	11 (0.028)*	2
Male	24 (44.4%)	0	2	4	2
Smokers	32 (58.2%)	2	2	7	2
Alcohol abuse	3 (5.5%)	0	0	1	0
Use of denture	4 (7.3%)	1	0	2	1
Diabetes	3 (5.5%)	1 (0.029)*	1 (0.029)*	2	0
Psychopharmacas	2 (3.6%)	1 (0.005)*	0	1	1 (0.019)*
Parotid	13 (24.1%)	3 (≤ 0.001)*	2	8 (0.002)*	1
Sterile extraction	5 (20.8%)	2 (≤ 0.001)*	2 (≤ 0.001)*	2	2 (0.003)*
Proton-pump inhibitors	4 (7.3%)	0	0	4 (< 0.001)*	0
Asthma medication	5 (9.1%)	1	0	2	0
Estrogen medication	7 (12.7%)	1	1	3	1
Reflux	4 (7.3%)	0	0	3 (0.026)*	1
Pulmonary diseases	11 (20%)	1	0	4	1
Hearth diseases	9 (16.4%)	0	0	2	1
Recurrent sialadenitis	23 (41.8%)	3 (0.036)*	3 (0.036)*	11 (≤ 0.001)*	2
Unilateral symptoms	46 (83.6%)	2	3	9	3
Stone in right side	33 (60.0%)	3	3	7	3
Papilar or ductal stone	39 (72.2%)	1	2	8	2

*n *number of patients, *SR* skin redness, *PD* pus drainage, *PI* post-operative infection

*Statistically significant means *p* < 0.05

A relation was found between pus drainage and reflux or use of proton-pump inhibitor (PPI) medication. In four reflux patients, three showed pus drainage (*p* = 0.026) which was also seen in all four patients using PPI (*p* < 0.001). The use of diabetes medication or psychopharmaca were also related with more severe symptoms (Table 1).

A total of 15 patients received peri- or post-operative antibiotics, mainly cephalexin (12) as monotherapy (9) or combined with amoxicillin, cephuroxime or metronidazole. Other administered antibiotics were amoxicillin with clavulanic acid and clindamycin used as monotherapy. Cases with antibiotic treatment were not statistically related with positive bacterial culture.

Among the 13 parotid stones, there were eight cases with pus drainage (*p* = 0.002) and three of them also showed skin redness (*p* ≤ 0.001). Of the 23 patients with recurrent sialadenitis 12 showed signs of worse evolution including pus drainage, fever, or skin redness (*p* ≤ 0.001) (Table 1).

Positive sonicated cultures were found in 45 salivary stones (82%), and among them, multiple organisms (two or more) were isolated in 33 stones (73% of the culture positive stones). In 25 stones (56%), oral bacteria was isolated. A total of 96 microorganisms were isolated. The main isolates were *Streptococcus mitis* [27 cases (28% of bacteria, 51% of patients and 60% of culture positive stones)] and *Streptococcus anginosus* [10 cases (10% of bacteria, 19% of patients and 22% of culture positive stones)]. No significant bacteriological difference was found between the salivary stones collected from parotid or submandibular gland. All isolated microorganisms are shown in Table 2.

**Table 2:** The microorganisms isolated from salivary stone

Bacteria	*n* (%)	Parotid	PI	SR	Fever	PD	SE	Swelling
*Streptococcus mitis/oralis*	27 (49.1%)	7	2	3	3	5	3	2
*S. anginosus*	10 (18.1%)	2	1	1	0	4	1	1
*Rothia* spp^a^	8 (14.5%)	1	0	0	0	2	1	1
*S. constellatus*	7 (12.7%)	1	3*	0	0	1	2	0
*S. gordonii*	6 (10.9%)	2	0	0	0	2	0	1
*S. aureus*	5 (9.1%)	0	2**	0	0	0	1	1
*Actinomyces viscosus*	5 (9.1%)	2	0	0	0	1	0	0
*Micrococcus* spp^b^	4 (7.3%)	1	0	0	0	1	0	0
*Bacillus cereus*	4 (7.3%)	1	0	1	2*	1	1	0
*S. epidermidis*	4 (7.3%)	1	1	0	0	1	1	0
*Haemophilus parainfluenzae*	3 (5.4%)	0	0	0	0	1	0	0
*Eikenella corrodens*	2 (3.6%)	0	1****	0	0	0	1	1
*Serratia marcescens*	1 (1.8%)	0	0	0	0	0	0	0
*S. sanguinis*	1 (1.8%)	0	0	0	0	0	0	0
*Escherichia coli*	1 (1.8%)	0	0	0	0	0	0	0
*Neisseria subflava*	1 (1.8%)	0	0	0	0	0	0	1***
*Gemella sanguinis*	1 (1.8%)	0	0	0	0	0	0	1***
*S. capitis*	1 (1.8%)	0	0	0	0	0	1*	0
*S. warneri*	1 (1.8%)	1	0	0	0	1	0	0
*Pseudomonas aeruginosa*	1 (1.8%)	0	0	0	0	0	0	0
*S. pneumoniae*	1 (1.8%)	1	0	0	0	1	0	0
*Propionibacterium acnes*	1 (1.8%)	0	0	0	0	0	0	0
*S. pyogenes*	1 (1.8%)	0	0	0	0	0	0	0

*n* number of salivary stones, *P-I* post-operative infection, *S-R* skin redness, *PD* pus drainage, *SE* sterile extraction

^a^ *Rothia mucilaginosa/Rothia dentocariosa*

^b^ *Micrococcus luteus/lylae*

*p ≤ 0.001; **p = 0.003; ***p = 0.004; ****p = 0.019

Considering only stones with positive culture, a total of 21 (47%) stones showed a high number of colony-forming units ( > 10^5^ CFU) in the culture for some microorganism. Biofilm formation was confirmed by fluorescence microscopy in the surface of 39 (71%) stones, and it was related with a positive culture in 37 (95%; *p* ≤ 0.001) and with high number of colony-forming units in 21 cases (57%; *p* = 0.004). Four patients suffered from post-operative infection, and in three of these cases (75%), biofilm was detected by microscopy and for all of them the culture was positive. A total of 23 patients showed recurrent sialadenitis and morphological evidence of bacterial biofilm or positive bacterial culture was detected in 19 (82.6%) and 20 (87%) of them respectively. All 15 patients with pus drainage had the presence of biofilm on the salivary stone (*p* = 0.004).

Clinical post-operative infections were related to *Eikenella corrodens* [one case (25% of cases with post-operative infection)] (*p* = 0.019), *S.aureus* [two cases (50%)] (*p* = 0.003), and *Streptococcus constellatus* [three cases (75%)] (*p* ≤ 0.001). The presence of *Gemella sanguinis* and *Neisseria subflava* was statistically related to swelling (*p* = 0.004 for both).

Use of antibiotic before surgery was related to *Bacillus cereus* and *Micrococcus luteus* (three cases and two cases from 15 cases where pre-operative antibiotic was used, respectively, 20% and 13%) (*p* = 0.034 and 0.024 respectively). When *Pseudomonas aeruginosa* or *Serratia marcescens* were isolated, there had been purulent discharge at the operation (*p* ≤ 0.001).

All cases of *H.parainfluenzae* were isolated from stones located in the ductal area (*p* ≤ 0.001). On the other hand, the presence of *Streptococcus anginosus* (four cases) and *Staphylococcus capitis* (one case) was related with the hilar or intraglandular area (*p* = 0.026 and 0.011 respectively). *Staphylococcus capitis* and *Eikenella corrodens* (20% both) were related to a sterile procedure (*p* ≤ 0.001 and 0.04 respectively).

Intestinal or food-contaminating bacteria such as *Actinomyces viscosus, Bacillus cereus, Serratia marcescens, *and* Escherichia coli* were isolated from six patients and in two cases related with reflux symptoms (*p* = 0.009) and in three cases with PPI medication (*p* ≤ 0.001).

## Discussion

We found a total of 23 different bacterial species associated with salivary stones. Over 80% of the stones were culture-positive after sonication, and, in addition, majority of these stones were culture-positive for more than one bacterium. Therefore, sonication of salivary gland stones could be a useful method for finding the etiology of the chronic infection and to detect the cases with potentially worse outcome. Our results are consistent with the previous studies, suggesting that bacterial biofilm leads to recurrent sialadenitis and, consequently, worse evolution of patients [16, 17].

In half of the stones, common oral bacteria were found. *Streptococcus oralis* and *S.anginosus* seemed to be the most common bacteria related to pre- or post-operative infections. However, it is difficult to determine the exact role of different microbes in stone formation and infection susceptibility, or if there is any role at all. There were no differences between microbes found in parotid and submandibular stones but some difference was observed when comparing the location of the stone. All cases of *H.parainfluenzae* were isolated from stones located in ductal area (*p* ≤  0.001), and presence of *Streptococcus anginosus* and *Staphylococcus capitis* were related to hilar or intraglandular location (*p* < 0.05).

When analyzing salivary stones, oral microbials are naturally found. They represent either a contaminant or the main pathogen adherent on the salivary stone. One method to distinguish between these two alternatives is the amount of colony-forming units found in the culture. In our study, the presence of oral bacteria was found in most of the stones, and this was not related with the location of the stone.

Biofilm formation was found in fluorescence microscopy in the surface of 71% of stones and it was statistically related with a positive culture (*p* ≤ 0.001) and with a high number of colony-forming units (*p* = 0.004). This might indicate that biofilm is a bacterial reservoir in the stone.

In general, high age, use of diabetes medication or psychopharmaca, and previous sialadenitis were predisposing factors for an infection. Somewhat surprisingly, there was a high number of post-operative infections among the cases where the stone was retrieved using a sterile technique. This may be related to the severity of the disease, complicated stone and almost always to an unsuccessful attempt to retrieve the stone first through an oral approach. Another explanation could be the contamination of sterile tissue (e.g., subcutaneous tissues) with non-sterile saliva.

An interesting observation was the positive correlation of the severity of sialadenitis with reflux and with use of PPI. Pus drainage was found in three out of four reflux patients and in all patients using PPI. Furthermore, in these cases intestinal or food-contaminating bacteria were isolated in the salivary stones. This may be related with the increased pH in the stomach due to the use of PPI. It promotes the survival of bacteria which may return to mouth with reflux.

The origin of salivary stones is unknown. It is easy to see the role of microbial biofilm in this process as its organic mass consists of glycoproteins and mucopolysaccharides. Biofilm tends to accumulate on an inactive surface such as a salivary stone and, thereafter, calcium deposition occurs in the same way as in the dental calculus [18]. In this study, we could show that microbial biofilm occurs in the majority of the salivary stones. However, considering the origin of the nidus, we end up with the chicken or the egg causality dilemma.

Parotid sialadenitis is a rare disease and parotid stones represent approximately 10–15% of the salivary stones [19]. Therefore, parotid stones are more difficult to study. To be able to increase statistical power considering parotid disease, we continued the collection of parotid stones longer. Our results show that parotid sialadenitis patients were older. This is in line with the known predisposing factors related to parotid stones such as secretory inactivity caused by the anticholinergic effect of some medication and dehydration related to the older age.

As a conclusion, bacterial biofilm was found to be related to more severe cases of sialadenitis. Between 75 and 100% of patients with clinical post-operative infections, recurrent sialadenitis or pus drainage showed bacterial biofilm.

The risk factors of sialolithiasis found in this study are high age, diabetes, and use of psychopharmaca. We also found an interesting correlation between both reflux symptoms and use of PPI medication and the severity of the sialadenitis.
